# Educational value of international and intercultural differences in prescribing: the international and interprofessional student-run clinic project

**DOI:** 10.1007/s00228-023-03465-9

**Published:** 2023-02-07

**Authors:** Michiel J. Bakkum, Erik M. Donker, Pietro Spitaleri Timpone, Charlotte A. M. Hagen, Milan C. Richir, Michiel A. van Agtmael, Fabrizio De Ponti, Jelle Tichelaar, on behalf of the Erasmus+ consortia European Prescribing Exam, European Open Platform of Prescribing Education and Clinical Pharmacology and Therapeutics Teach the Teacher program, and the EACPT Education Working Group, Robert Likic, Ylva Böttiger, Thierry Christiaens, Cornelis Kramers, João N. Costa, Emilio J. Sanz, Paraskevi Papaioannidou, Joost Piët, Lorena Dima, Jeroen van Smeden, Jitka Rychlícková, Floor van Rosse, Susanna M. Wallerstedt, Markus Schwaninger, Yves-Marie Pers, David J. Brinkman, Carla Sans-Pola, Jamie J. Coleman, Romaldas Maciulaitis, Bogdan Ionel Tamba

**Affiliations:** 1 Department of Internal Medicine, Amsterdam UMC, Unit Pharmacotherapy, Vrije Universiteit Amsterdam, De Boelelaan 1117, 1081HV Amsterdam, The Netherlands; 2 Research and Expertise Centre in Pharmacotherapy Education (RECIPE), Amsterdam, The Netherlands; 3 Department of Medical and Surgical Sciences, Alma Mater Studiorum, Pharmacology Unit, University of Bologna, Bologna, Italy; 4 Department of Surgery, University Medical Center Utrecht, Utrecht, The Netherlands

**Keywords:** Clinical pharmacology, Education, Interprofessional, Undergraduate

Treatment guidelines differ significantly, not only between Europe and North America but also among European countries [1–4]. Reasons for these differences include antimicrobial resistance patterns, accessibility to and reimbursement policies for medicines, and culturally and historically determined prescribing attitudes. The European Association of Clinical Pharmacology and Therapeutics’ Education Working Group has launched several initiatives to improve and harmonize European pharmacotherapy education, but international differences have proven to be a major barrier to these efforts [5–7]. While we have taken steps to chart these differences [6, 8], it will probably not be possible to fully resolve them. Rather than viewing these differences as a barrier, we should perhaps see them as an opportunity for intercultural learning by providing students and teachers a valuable lesson in the context-dependent nature of prescribing medication and the different interpretations of evidence-based medicine. Here, we extend our experience with interprofessional student-run clinics [9, 10], to report on our first experiences with the “International and Interprofessional Student-run Clinic.”

We organized three successful video meetings with medical and pharmacy students of the Amsterdam UMC, location VU University (the Netherlands), and the University of Bologna (Italy). During these meetings, one of the students presented a real-life case of a patient on polypharmacy. Then, in a 45-min session, the students split into smaller groups (break-out rooms) to review the patient’s medication, using the prescribing optimization method and STOPP/START criteria [11, 12]. The teachers rotated between the different rooms and assisted the students when necessary. Teachers and students reconvened for 60 min for debriefing, with students presenting their findings and suggestions to revise the medication list and teachers stimulating discussion and indicating how they would alter the medication list. Participation was voluntary, and the meetings were held in the evenings to accommodate students in clinical rotations.

Third-to-final-year medical and pharmacy students participated in the three meetings (*n* = 17, *n* = 20, *n* = 12, respectively). They reported learning a lot from each other, gaining an international and interprofessional perspective. Moreover, they learned to always consider the patient’s perspective, that evidence-based medicine is context-dependent, and that guidelines should be adapted to the patient’s situation.

There were marked differences in prescribing guidelines and the use and accessibility of medicines (Table 1). In both countries, national societies develop guidelines based on international standards, but in the Netherlands, most hospitals have their own local guidelines as well, resulting in different prescribing preferences (e.g., use of different low-molecular-weight heparins). Differences in prescribing preferences exist in Italy, but are based on regional, not local, formularies. For instance, the Emilia-Romagna region has a periodically revised formulary, whereas the Lombardy region does not. Moreover, while in the Netherlands, physicians are allowed to prescribe almost all marketed drugs, even if their costs are not always reimbursed; in Italy, certain drugs may only be prescribed by a specialist (e.g., new glucose-lowering drugs, until January 2022) [13].

**Table 1:** Examples of differences between clinical practice guidelines and the use of medicines in the Netherlands and Italy

**Subject**	**Dutch situation**^a^	**Italian situation**^b^
Acetylsalicylic acid	Almost exclusively used in low dosages as anti-platelet therapy (41.7 DDD/1000 inhabitants/day)	Commonly used as over-the-counter nonsteroidal anti-inflammatory drug^c^ and as anti-platelet therapy (46.1 user DDD/1000 inhabitants/day)
Direct oral anticoagulants^d^	Commonly prescribed by medical specialists and general practitioners (18.6 DDD/1000 inhabitants/day) [16]	Almost exclusively prescribed by medical specialists due to prescribing restrictions that were lifted only recently (15.2 DDD/1000 inhabitants/day) [17]
Vitamin D supplements	Advised as primary prevention for osteoporosis in postmenopausal women and men aged 70 years or older (65.3 DDD/1000 inhabitants/day). Selective reimbursement since 2019, possibly leads to increase of over-the-counter use^c^. Per 2023 no reimbursement at all (over-the-counter use promoted)	In adults, reimbursement was recently restricted to selected conditions because of its potentially inappropriate use (142.9 DDD/1000 inhabitants/day)
Gastric ulcer prophylaxis^e^	Very directive guidelines intended to avoid under- and overuse of PPIs (126.8 DDD/1000 inhabitants/day)	Guidelines aimed at preventing gastric ulcers/bleeding, but not at reducing overuse (79.8 DDD/1000 inhabitants/day)
Osmotic laxatives with opioids^f^	Prophylactic treatment with laxatives is advised when opioids are started (18.9 DDD/1000 inhabitants/day)	Laxatives are reactively given when constipation develops (2.2 DDD/1000 inhabitants/day)
Type II diabetes mellitus	SU derivates^g^ remain second-line treatment (for patients without prior cardiovascular or renal disease) (24.6 DDD/1000 inhabitants/day)	SU derivates virtually abandoned since the introduction of SGLT2-inhibitors and GLP1-agonists (7.1 DDD/1000 inhabitants/day)
Angina pectoris prophylaxis	Long-acting nitrates usually administered orally	Long-acting nitrates usually administered either via skin patches or oral tablets

*DDD* defined daily dose, *PPI* proton pump inhibitor, *SU* sulfonylurea, *SGLT2* sodium/glucose cotransporter-2, *GLP* glucagon-like peptide-1

^a^Data based on figures of the Dutch National Health Care Institute [18]

^b^Data based on figures of OSMED report - Italian Medicines Agency (AIFA) [19]

^c^No data about over-the-counter use available

^d^Apixaban, rivaroxaban, edoxaban, dabigatran

^e^Omeprazole, pantoprazole, lansoprazole, rabeprazole, esomeprazole

^f^Macrogol, lactulose

^g^Glibenclamide, tolbutamide, gliclazide, glimepiride

These first experiences with international and interprofessional case discussions during the COVID pandemic have taught us that geographical distance no longer needs to be an obstacle to organizing educational events. This, together with the earlier finding that an interprofessional student-run medication review program could optimize pharmacotherapy and reduce adverse drug events, is a promising development [14, 15]. We intend to expand the scope of these case discussions by including different universities and larger (intra-curricular) assignments. Five other European universities monitored the last two meetings and want to participate in future meetings. The European Open Platform of Prescribing Education (EurOP^2^E) provides a meeting place for international teachers wishing to organize such meetings and helps to facilitate them. Interested teachers can apply via www.prescribingeducation.eu.

## Data Availability

Not applicable.

## References

[1] van der Meer P, Gaggin HK, Dec GW (2019). ACC/AHA versus ESC guidelines on heart failure: JACC guideline comparison. J Am Coll Cardiol.

[2] Youssef A, Vermeulen N, Lashley EELO, Goddijn M, van der Hoorn MLP (2019). Comparison and appraisal of (inter)national recurrent pregnancy loss guidelines. Reprod Biomed Online.

[3] Mohan GC, Lio PA (2015). Comparison of dermatology and allergy guidelines for atopic dermatitis management. JAMA Dermatol.

[4] Becker M, Breuing J, Nothacker M, Deckert S, Brombach M, Schmitt J, Neugebauer E, Pieper D (2019). Guideline-based quality indicators—a systematic comparison of German and international clinical practice guidelines. Implement Sci.

[5] Bakkum MJ, Richir MC, Papaioannidou P, Likic R, Sanz EJ, Christiaens T, Costa JN, Mačiulaitis R, Dima L, Coleman J, Tichelaar J, van Agtmael MA (2021). EurOP(2)E - the European Open Platform for Prescribing Education, a consensus study among clinical pharmacology and therapeutics teachers. Eur J Clin Pharmacol.

[6] Brinkman DJ, Tichelaar J, Mokkink LB, Christiaens T, Likic R, Maciulaitis R, Costa J, Sanz EJ, Maxwell SR, Richir MC, van Agtmael MA, Education working group of the European Association for Clinical P, Therapeutics, its affiliated Network of Teachers in P (2018). Key learning outcomes for clinical pharmacology and therapeutics education in Europe: a modified Delphi study. Clin Pharmacol Ther.

[7] Donker EM, Brinkman DJ, Richir MC, Papaioannidou P, Likic R, Sanz EJ, Christiaens T, Costa JN, De Ponti F, Böttiger Y, Kramers C, van Agtmael MA, Tichelaar J (2022). The European Prescribing Exam: assessing whether European medical students can prescribe rationally and safely. Eur J Clin Pharmacol.

[8] Donker E, Brinkman D, Richir M, Papaioannidou P, Likic R, Sanz EJ, Christiaens T, Costa J, De Ponti F, Gatti M, Böttiger Y, Kramers C, Garner S, Pandit R, van Agtmael M, Tichelaar J (2021) European list of essential medicines for medical education: a protocol for a modified Delphi study. BMJ Open 11(5):e045635. 10.1136/bmjopen-2020-045635 10.1136/bmjopen-2020-045635 PMC8098946 33947736

[9] Schutte T, Tichelaar J, van Agtmael M (2016). Learning to prescribe in a student-run clinic. Med Teach.

[10] Dekker RS, Schutte T, Tichelaar J, Thijs A, van Agtmael MA, de Vries TP, Richir MC (2015). A novel approach to teaching pharmacotherapeutics–feasibility of the learner-centered student-run clinic. Eur J Clin Pharmacol.

[11] Drenth-van Maanen AC, van Marum RJ, Knol W, van der Linden CMJ, Jansen PAF (2009). Prescribing optimization method for improving prescribing in elderly patients receiving polypharmacy. Drugs Aging.

[12] O'Mahony D, O'Sullivan D, Byrne S, O'Connor MN, Ryan C, Gallagher P (2014). STOPP/START criteria for potentially inappropriate prescribing in older people: version 2. Age Ageing.

[13] Agenzia Italiana del Farmaco (2022) NOTA 100. In: ed. https://www.aifa.gov.it/documents/20142/1728125/nota-100.pdf

[14] Sultan R, van den Beukel TO, Reumerman MO, Daelmans HEM, Springer H, Grijmans E, Muller M, Richir MC, van Agtmael MA, Tichelaar J (2022). An interprofessional student-run medication review program: the clinical STOPP/START-based outcomes of a controlled clinical trial in a geriatric outpatient clinic. Clin Pharmacol Ther.

[15] Reumerman MO, Richir MC, Sultan R, Daelmans HEM, Springer H, Grijmans E, Muller M, van Agtmael MA, Tichelaar J (2022) An inter-professional student-run medication review programme. Reducing adverse drug reactions in a memory outpatient clinic: a controlled clinical trial. Expert Opin Drug Saf 1–10. 10.1080/14740338.2022.2069748 10.1080/14740338.2022.2069748 35469517

[16] de Jong LA, Koops M, Gout-Zwart JJ, Beinema MJ, Hemels MEW, Postma MJ, Brouwers J (2018). Trends in direct oral anticoagulant (DOAC) use: health benefits and patient preference. Neth J Med.

[17] Agenzia Italiana del Farmaco (2020) NOTA 97. https://www.aifa.gov.it/documents/20142/1728116/nota-97.pdf

[18] Zorginstituut Nederland / GIPdatabank. https://www.gipdatabank.nl/. Accessed Dec 2022

[19] Italian Medicines Agency (2022) The medicines utilisation monitoring centre. National Report on Medicines use in Italy. Year 2021

